# Exercise training and chronic kidney disease: characterization and bibliometrics of citation classics of clinical intervention trials

**DOI:** 10.1080/0886022X.2024.2349187

**Published:** 2024-05-09

**Authors:** Fan Zhang, Shan Liu, Yan Bai, Liuyan Huang, Yifei Zhong, Yi Li

**Affiliations:** Department of Nephrology A, Longhua Hospital Shanghai University of Traditional Chinese Medicine, Shanghai, China

**Keywords:** Bibliometric analysis, exercise training, chronic kidney disease, citation classics

## Abstract

**Background:**

Exercise research targeting chronic kidney disease (CKD) has been conducted for more than 30 years, and the benefits of exercise for CKD patients have been progressively demonstrated. This study analyzes citation classics on clinical intervention trials on exercise training and CKD to describe the research landscape and hotspots through bibliometric analysis.

**Methods:**

To identify clinical trials of exercise training interventions for CKD with more than 100 citations from the Web of Science Core Collection database. Extracted bibliometric information, participant information, and study characteristics of the included articles. The total citations, annual average citations, publication of year, author keywords, and study-related data were bibliometric analyzed and described using Excel 2019 and VOSviewer software.

**Results:**

A total of 30 citation classics were included, with a total citation frequency of 102 to 279 (mean ± standard deviation: 148.4 ± 49.4). The *American Journal of Kidney Diseases* (*n* = 7) published the most (*n* = 7) classic citations in the field of CKD exercise research, and the *Journal of the American Society of Nephrology* was the most cited. The hotspot of research around CKD and exercise training interventions focused on population (hemodialysis and end-stage renal disease), exercise type (resistance training, yoga, and leg-cycling), and outcomes (cardiovascular indices, physical performance, psychological status, kidney function, physical activity). Reported dropout rates ranged from 0.0% to 47.4%.

**Conclusion:**

A bibliometric analysis of citation classics on exercise training and CKD highlights the potential benefits of exercise as a non-pharmacological therapy for patients with CKD, as well as developments and hotspots in the field.

## Introduction

In recent years, CKD has become the ninth leading cause of death in high-income countries, and its incidence is increasing yearly, with a trend toward younger people [1]. The global loss of life expectancy due to CKD is expected to double by 2040, representing a formidable challenge for healthcare and health systems [2].

Most CKD patients have very low exercise endurance, although the exact mechanism is not fully understood, the catabolic state, mitochondrial dysfunction, cardiovascular complications, mineral-skeletal disorders, and anemia associated with kidney disease result in impaired muscle function and decreased maximal oxygen capacity [3,4]. Exercise intolerance is a strong prognostic factor associated with mortality, independent of renal function [5]. Therefore, increasing or maintaining exercise endurance is a key factor in improving the quality of life of patients with CKD.

Renal rehabilitation, a comprehensive multidisciplinary program for patients with renal dysfunction, combines exercise training, dietary therapy, hydration management, medication, patient education, and spiritual support over time [6,7]. The benefits of exercise training as a core component of renal rehabilitation have been progressively demonstrated over the past 30 years [8–10]. In recent years, it has been argued that, for CKD patients, moderate exercise not only does not exacerbate the deterioration of renal function but also increases exercise tolerance and quality of life, improves inflammatory response [11,12], and protein isomerization can be prevented by exercise even with a low-protein diet [13]. Thus, exercise should not be unduly restricted for CKD patients. Additionally, a growing body of evidence has led various national renal societies to develop clinical practice guidelines for exercise and lifestyle in CKD [14–16].

In this context, an international group of researchers and clinicians met in Chicago in November 2016 as the Global Renal Exercise Working Group to discuss research priorities related to exercise in CKD [17]. They meet regularly to promote collaborative interdisciplinary research and innovation to develop practical strategies to increase physical activity in people with CKD. With the development of the “Exercise is Medicine” concept, exercise intervention has received increasing attention in clinical trials on the prognosis of CKD patients [18].

Citation refers to an article (citation) citing another article (cited) as a reference. The number of citations is a metric of an article’s impact in the scientific community and the basis for generating a journal’s Impact Factor (IF) [19]. Eugene Garfield proposed the term “citation classic” in 1955 to identify the most cited articles in the Institute for Scientific Information (ISI) Web of Knowledge (now known as Web of Science) database [20]. In most fields, articles with more than 100 citations are considered citation classics [20]. Reviewing the most cited articles (so-called “citation classics”) can provide interesting information about scientific advances and research trends in specific subject areas [21].

The primary objective of this study was to identify the citation classics with a citation number higher than 100 in clinical trials on exercise interventions for patients with CKD and to describe their main characteristics. The secondary objective was to visualize and analyze the keywords using the VOSviewer software, to deepen nephrology health caregivers’ understanding of the current status and hot spots of the research, and to provide a reference for future directions.

## Materials and methods

### Search strategy

As a source of publications, we chose the Web of Science Core Collection (SCI-Expanded 1980), which contains more than 20,000 peer-reviewed, high-quality scholarly journals in more than 250 medical, social science, and humanities disciplines worldwide and is widely used for bibliometric analyses. In addition, the database provides the author, country, and keywords of each paper, which is essential for the current study.

The search strategy was TS=(“chronic renal insufficiency” OR “chronic kidney insufficiency” OR “chronic kidney failure” OR “chronic renal failure” OR “chronic kidney disease” OR “chronic renal disease” OR “predialysis” OR “pre-dialysis” OR “end-stage kidney” OR “end-stage renal” OR “endstage kidney” OR “endstage renal” OR “dialysis” OR “hemodialysis” OR “hemodialysis” OR “hemodiafiltration” OR “haemodiafiltration” OR “hemofiltration” OR “haemofiltration” OR “renal transplantation” OR “kidney grafting” OR “kidney transplantation”) AND TS=(“exercise” OR “endurance training” OR “resistance training” OR “muscle training” OR “physical activity” OR “Tai Chi” OR “Tai-ji” OR “Tai Ji Quan” OR “Taiji” OR “Baduanjin” OR “Qi gong” OR “Yoga” OR “walking” OR “swimming” OR “running” OR “jogging” OR “interval training” OR “Pilates”).

### Publication selection and data extraction

First, the type of literature was restricted to “article” from the initial 6,812 retrieved literature, and then the title and abstract of each article were independently reviewed by two authors for relevance to CKD and exercise interventions. Then, “citation classics” were selected for bibliometric analysis based on the total citations ranked with a cutoff value 100. Finally, 30 articles were included in this study (Figure S1).

Thirty citation classics were reviewed, and the following information was extracted: 1) metrological information: authors, publication year, journal, IF (2022), total citations, and annual average citations; 2) stage of CKD, sample size of enrollment and number of dropouts to calculate dropout rates; 3) study characteristics: study design, exercise type, duration of intervention, and primary outcomes.

### Visualization

All information and data from each article were input into Microsoft Excel 2019 and VOSviewer (version 1.6.15). VOSviewer is a Java-based bibliometric network analysis software for bibliometric data for unimodal undirected networks focusing on visualizing scientific knowledge [22]. Co-occurrence analysis is performed on keywords to identify research hotspots.

### Statistical analysis

Data were analyzed using descriptive statistics without statistical significance tests.

## Results

**Figure 1.**
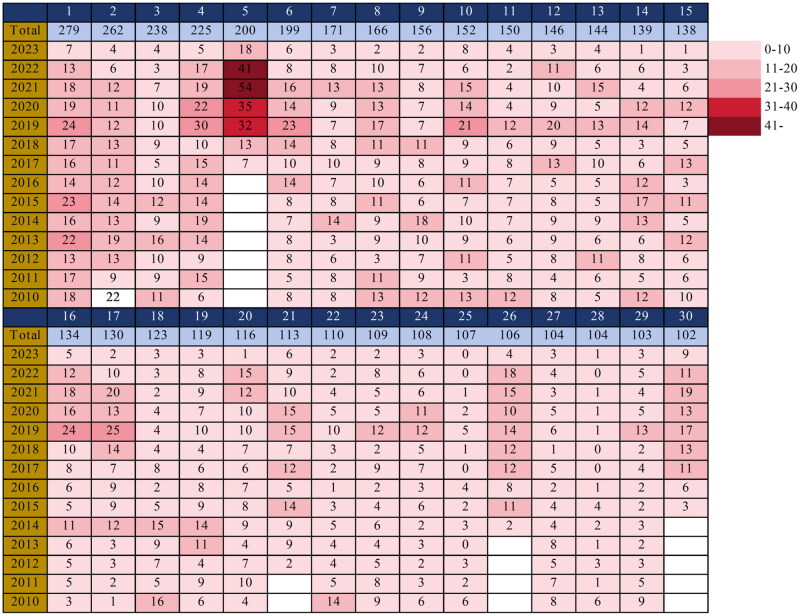
Hot-map of total citations for classic citations in clinical trials of exercise training and CKD. Note: we coded classical citations from highest to lowest total number of citations, consistent with the numbering in Table S1.

The total citations for citation classics of CKD exercise intervention trials ranged from 102 to 279 (mean ± standard deviation [SD]: 148.4 ± 49.4), and the annual average citations per clinical trial ranged from 2.82 to 28.57 (mean ± SD: 8.4 ± 4.8). When comparing the annual citations ranking to total citations, the change in position ranged from +0.0 to +8.0 (Figure S2). The highest total citation was obtained for the clinical trial published by Johansen KL et al. (*n* = 279), whereas Manfredini F et al. had the highest annual average citation (*n* = 28.57) (see Table S1). We plotted a heat map (2010 to 2023) of classical citations by total and annual average citations (Figure 1).

Thirty citation classics were published in 14 journals with IFs ranging from 1.8 to 39.2 (mean 8.8 ± 10.2; median 4.0) (Table 1). The *American Journal of Kidney Diseases* (*n* = 7) published the most (*n* = 7) citation classics in CKD exercise research, and the *Journal of the American Society of Nephrology* was the most cited.

**Table 1.** Number of classic citations by journals.

Journals	Number of articles	2022 IF	Citation	JCR Category *	Category Quartile
American Journal of Kidney Diseases	7	13.2	1051	Urology & Nephrology (6/88)	Q1
Journal of the American Society of Nephrology	6	13.6	1054	Urology & Nephrology (5/88)	Q1
Nephrology Dialysis Transplantation	5	6.1	786	Transplantation (4/26) / Urology & Nephrology (10/88)	Q1/Q1
Nephron	2	2.5	218	Urology & Nephrology (23/52)	Q2
Kidney International	1	19.6	104	Urology & Nephrology (3/88)	Q1
American Journal of Cardiology	1	2.8	104	Cardiac & Cardiovascular Systems (80/142)	Q3
Annals of Internal Medicine	1	39.2	171	Medicine, General & Internal (6/167)	Q1
Archives of Physical Medicine and Rehabilitation	1	4.3	146	Rehabilitation (8/68) / Sport Sciences (13/87)	Q1
Clinical Physiology and Functional Imaging	1	1.8	109	Physiology (64/79)	Q4
Clinical Rehabilitation	1	3	152	Rehabilitation (16/68)	Q1
Complementary Therapies in Medicine	1	3.6	116	Integrative & Complementary Medicine (10/28)	Q2
International Journal of Cardiology	1	3.5	103	Cardiac & Cardiovascular Systems (62/142)	Q2
Journal of Rehabilitation Medicine	1	3.5	199	Rehabilitation (11/68) /Sport Sciences (19/87)	Q1
Transplantation	1	6.2	144	Immunology (45/161)/Surgery (12/212)/ Transplantation (3/26)	Q2/Q1/Q1

**Figure 2.**
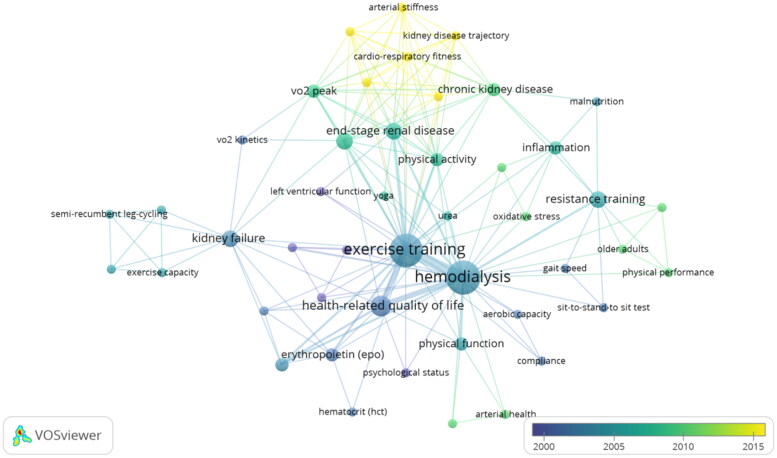
Keyword co-occurrence analysis.

Based on the keywords co-occurrence (Figure 2), the network mapping showed that the research hotspots around CKD and exercise interventions mainly focused on population (hemodialysis and end-stage renal disease), exercise type (resistance training, yoga and leg-cycling), outcomes (cardiovascular indices [arterial stiffness; left ventricular function, arterial health], physical performance [cardiorespiratory fitness, sis-to-stand sit test, gait speed, aerobic capacity], psychological status, kidney function, physical activity).

**Figure 3.**
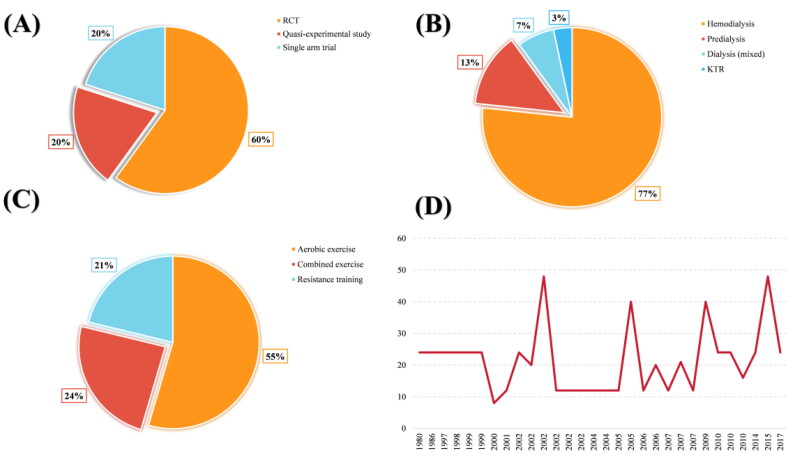
Study characteristics of 30 classic citations. *Note:* (A) Over half of the 30 citation classics were randomized controlled trials, with six single-arm trials and quasi-experimental studies, respectively. (B) For the study population, more than 3/4 were hemodialysis-dependent CKD patients, only one study recruited kidney transplant recipients, and no separate clinical studies included peritoneal dialysis patients. (C) Regarding exercise type, aerobic exercise was reported in 55% of the studies, with a comparable proportion of combined exercise and resistance training. (D) Thirty citation classics reported exercise durations that fluctuated considerably between years, with the longest being 48 weeks.

Over half of the 30 citation classics were randomized controlled trials, with six single-arm trials and quasi-experimental studies, respectively (Figure 3(A)). For the study population, more than third-fourth were hemodialysis-dependent CKD patients, only one study recruited kidney transplant recipients, and no separate clinical studies included peritoneal dialysis patients (Figure 3(B)). Regarding exercise type, aerobic exercise was reported in 55% of the studies, with a comparable proportion of combined exercise and resistance training (Figure 3(C)). Thirty citation classics reported exercise durations that fluctuated considerably between years, with the longest being 48 weeks (Figure 3(D)).

**Figure 4.**
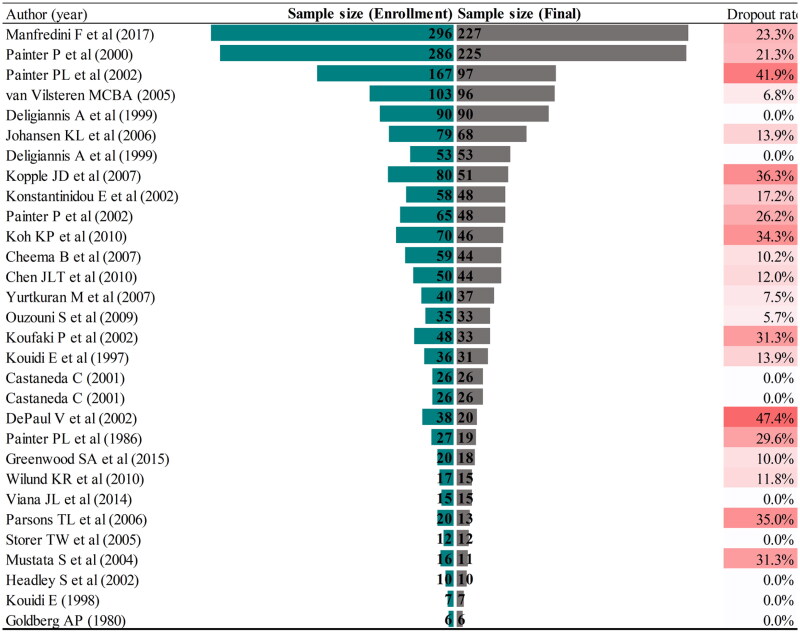
Sample size and dropout rate of 30 classic citations.

The participants recruited for the 30 citation classics reports ranged from 6 to 296, and the dropout rates ranged from 0.0% to 47.4% (Table S1 and Figure 4). Reported reasons for dropout include death, transplant, not wishing to continue, and medical illness.

## Discussion

Landmarks or significant clinical trials are often the basis for clinical practice guidelines. Ideally, healthcare professionals would incorporate content from these guidelines or trial results into patients’ routine care as a structural element of quality improvement. This study further analyzes the published article “Exercise-Based Renal Rehabilitation: A Bibliometric Analysis From 1969 to 2021” [23]. Citation classics in clinical trials of exercise interventions in CKD were selected and described along with their main characteristics. The study reflects the status, hot topics, and frontiers of the clinical implementation of exercise as an adjunctive therapy for patients with CKD.

Citation analysis is the branch of bibliometry investigating the citation an article receives [19]. The total citations are widely used to assess individual articles’ impact and measure the quality of journals [20]. This study summarizes several characteristics of high-impact research on exercise interventions in CKD: 1) Some articles with high total citations but low annual citations may be historically significant findings. 2) These citation classics were primarily published in high-quality journals, which is not surprising; high-impact articles tend to be published in high-level journals, increasing the prestige of these journals [24]. 3) Clinical trials of exercise interventions for patients with CKD are gradually moving away from physical performance to cardiovascular event outcomes. And 4) most high-impact articles focus on patients treated with hemodialysis, which allows insufficient evidence for pre-dialysis CKD patients, peritoneal dialysis patients, and kidney transplant recipients. 5) Dropout rates of exercise interventions in CKD patients remain a concern.

The citations for citation classics in CKD exercise interventions ranged from 102 to 279, comparable to bibliometric results for similar topics in heart failure [25] and chronic obstructive pulmonary disease [26]. There is a view that the total citations may be influenced by the publication year, i.e. papers published earlier are cited more often than those published more recently [27]. Therefore, we analyzed the annual average citations of articles. This approach provides a perspective on the current impact of an article. The annual citations significantly changed the ranking of classic articles. For example, Johansen KL et al. [28] published a clinical trial in 2006, which was published earlier and had the highest total citations. This suggests that the study was essential and highly relevant to historical and current concerns. Although Manfredini F et al. [29] had the fifth-highest total citations, it had the highest average annual citations, suggesting that the trial has received more attention. Another example is the study by Greenwood SA et al. [30], which was the last in total citations but the fourth highest in annual average citations. Articles with high total citations but low annual citations are more likely to be historically essential and may not reflect current impact [31]. In contrast, articles with high total and annual citations may be the current situation that researchers should be concerned about. It is important to note that IF or citations are not an indicator of the quality of scientific research but rather a measure of recognition. In other words, the citations to an article should not be considered equivalent to its importance [32].

In terms of published journals, more than 2/3 (*n* = 21) of the citation classics for exercise interventions in CKD were published in Urology & Nephrology discipline-specific journals, which is not surprising; after all, nephrology healthcare professionals have begun to pay attention to exercise rehabilitation for CKD patients. We also note that articles in this field have been published in non-specialty journals such as Cardiac & Cardiovascular Systems, Rehabilitation, and Integrative & Complementary Medicine, demonstrating the multidisciplinary nature of exercise management in CKD. Patients with CKD, even those in the early stages of CKD who have not reached renal failure, are at high risk for cardiovascular disease, and assessing comorbidities, nutritional levels, psychological status, and exercise capacity in patients with CKD is necessary [33]. Ideally, a multidisciplinary team of professionals, including clinicians (nephrologists, cardiologists, and endocrinologists), exercise physiologists, dietitians, and nurses, must be formed to develop an exercise prescription and supervise regular participation in exercise, which will lead to better management of exercise rehabilitation in CKD patients [34,35].

The keywords of 30 citation classics were analyzed for co-occurrence by VOSviewer to describe the research hotspots. It is not difficult to see that the outcome of concern in clinical trials of exercise interventions for CKD patients is gradually shifting from physical performance to cardiovascular events. Physical inactivity and decreased exercise endurance are prevalent in CKD patients, and they interact to form a vicious cycle, exacerbating muscle atrophy and cardiorespiratory impairments and affecting patients’ prognosis and quality of life [36]. Therefore, over the past 20 years, the results of exercise interventions for CKD have focused on physical performance, including cardiorespiratory fitness (assessing exercise capacity), sis-to-stand sit test (assessing lower extremity muscle strength), and gait speed (assessing mobility). These results also resulted in a strongly recommended level of evidence [15,16].

Cardiovascular disease is a major complication of CKD, and most patients with CKD die of cardiovascular disease before they develop renal failure [37]. Considering this, the focus of trial results shifted to outcomes prioritized by renal care stakeholders, including patients, particularly the cardiovascular disease burden [4]. Exercise-based cardiac rehabilitation is a well-established adjunctive therapy that reduces mortality, and all-cause hospitalization in patients at low risk for coronary heart disease and is a Class I recommended treatment [38]. As drivers of cardiovascular risk are comparable, it is reasonable to assume that the benefits of cardiac rehabilitation also apply to patients with mild to moderate CKD [4]. Results from a recent randomized controlled trial published in *Kidney International* demonstrated that a 6-month intradialytic cycling exercise program significantly reduced participants’ left ventricular mass and showed no increase in either ventricular ectopic beats or complex ventricular arrhythmias [39]. Chowdhury R et al. [40] concluded that this study fills a critical gap in the field and adds to the growing body of evidence demonstrating the beneficial effects of exercise on health outcomes in dialysis-dependent CKD patients. The study was published in 2021, and the article has received 20 citations as of the search date.

Regarding study characteristics, most of the literature related to exercise interventions in CKD, in terms of citation classics and current published studies, has been directed at hemodialysis patients. In contrast, relatively few studies have been conducted in pre-dialysis and kidney transplant recipients [41], and trials recruiting peritoneal dialysis patients are even more lacking [42]. The reasons for this are twofold: 1) hemodialysis patients spend a few hours on dialysis three days a week and usually have little to do during their treatment, so exercise in dialysis is considered relatively convenient and time-saving. The literature also reports low dropout rates from trials of intradialytic exercise. Unfortunately, almost all hemodialysis exercise trials rely on a “one-size-fits-all” prescription for stationary bicycle exercise during dialysis. 2) Peritoneal dialysis usually needs to be performed at home and is relatively frequent, possibly several times daily. This may limit patients’ time and energy to engage in exercise. Studies based on exercise interventions have reported benefits for patients with end-stage renal disease treated with hemodialysis [8]; however, there are few studies explicitly involving patients on peritoneal dialysis, and there is a lack of well-designed randomized controlled trials that would allow for significant and valid evidence-based research on exercise and peritoneal dialysis patient-oriented outcome measures [42]. The first exercise guideline focusing on peritoneal dialysis patients, published in 2019 by the International Society for Peritoneal Dialysis in conjunction with the Global Renal Exercise Network, also reported a severe lack of available evidence [43].

Implementing exercise programs that produce benefit maximization in CKD patients remains a daunting challenge, but there are reasons for optimism. In the classic citation above, most participants were able to benefit from the exercise intervention. Thus, the disparate results in many studies may manifest that large, long-term exercise intervention studies in CKD patients are challenging to conduct, rather than evidence that exercise is ineffective. What can be done to make exercise interventions more effective for more patients? One crucial reason is high adherence and low dropout rates. The 30 clinical trials in this study reported the highest dropout rate of nearly 50%; in other words, almost half of all the participants did not follow the established protocol during the study period. The results of a retrospective study [44] showed that ‘non-completers’ (successful completion of a pragmatic renal rehabilitation program more than 50%) of renal rehabilitation had a 1.6-fold (95% confidence interval 1.00–2.58) greater risk of a combined event (*p* = .048). It is important to note that the calculation of dropout rates included non-motivated dropouts due to death, transplantation, or other sudden illness, and fewer participants dropped out of the trial due to a “lack of interest in exercise.” Improving patient compliance and reducing dropout is essential to ensure that exercise interventions are delivered in practice.

Based on the above analysis, several recommendations can be made for future research. The first recommendation is that adapting previously validated exercise-related hemodialysis trials to a representative sample of the peritoneal dialysis population may be a possible future course of action. After all, there is currently little knowledge surrounding exercise in peritoneal dialysis patients. A recent international-wide survey showed that most clinical professionals agree that organized exercise programs benefit patients undergoing peritoneal dialysis treatment and that more exercise could be performed [45]. Second, the cardiovascular disease burden remains a focal point for patients with CKD, and the conflicting results of exercise interventions on cardiovascular markers make support for the claim that “exercise improves cardiovascular prognosis in CKD” surprisingly weak [46]. Third, integrating the role of environmental and social factors may enrich the results of future studies. This question focuses on considering individuals who cannot exercise at home, and the preferred option may be a health clinic [47], which would require consideration of integrating exercise areas into nephrology wards or dialysis centers to facilitate the implementation of exercise routines.

There are several limitations of this study that are of concern. First, although we have used CKD-related terms for our search strategy where possible, we may still have missed some citation classics related to our subject. Second, this study was not a systematic review, and only the Web of Science database was selected for this analysis, and the high-impacted literature included in other databases, such as PubMed and Scopus, may have been omitted. Third, an article’s total citations accumulate over time, meaning older publications will undoubtedly receive more citations than newer ones. The impact of more recent publications may be underestimated due to the short time citations are generated. In addition, some potential factors, such as omission bias (i.e. the tendency not to cite competitors or sources that contradict one’s results) and self-citation, may indirectly increase the citations to an article [48]. Fourth, the list of most cited papers is dynamic and changes over time as the field progresses. Regularly updating the classic citations on exercise interventions for patients with CKD is something that future researchers should consider.

## Conclusion

Our study details 30 citation classics of exercise intervention trials in CKD, which provide a deeper understanding of current research hotspots and prospects. Overall, research in exercise as an adjunctive therapy for CKD patients has made tremendous progress, gradually clarifying the benefits for hemodialysis patients. It is reasonable to believe that higher-quality clinical trials will complement the evidence of the therapeutic role of exercise in different CKD stages in the future.

## Supplementary Material

Supplemental Material

## Data Availability

The data covered herein have been included in the manuscript and supplementary materials.
