# Supplementary material for: Exercise training and chronic kidney disease: characterization and bibliometrics of citation classics of clinical intervention trials
Source: Ren Fail. 2024 May 9;46(1):2349187. doi: 10.1080/0886022X.2024.2349187 (PMC11085942; doi:10.1080/0886022X.2024.2349187)
Supplement: Supplemental Material [file IRNF_A_2349187_SM2829.docx]

**Exercise training and chronic kidney disease: characterization and bibliometrics of citation classics of clinical intervention trials**

**Supplementary Material**

**Content**

[Table S1 The characteristic of included 30 citation classics 2](#_Toc144665101)

[Figure S1 Flow chart of literature screening. 11](#_Toc144665102)

[Figure S2 Dumbbell plots of total citations and annual average citations for 30 classic citations 12](#_Toc144665103)

[Reference 13](#_Toc144665104)

Table S1 The characteristic of included 30 citation classics

| Rank | First author (Year) [Ref.] | Title | Study design | Sample size (Final) | Dropout rate | Population | Intervention | Primary outcomes | Duration | Total citations | Annual average citations |
| --- | --- | --- | --- | --- | --- | --- | --- | --- | --- | --- | --- |
| 1 | Johansen KL (2006) [1] | Effects of resistance exercise training and nandrolone decanoate on body composition and muscle function among patients who receive hemodialysis: A randomized, controlled trial | RCT | 79 (68) | 13.9% | HD | Placebo injections vs. weekly nandrolone decanoate vs. intradialytic resistance training+placebo injections vs. intradialytic resistance training+weekly nandrolone decanoate | 1) Lean body mass measured by dual-energy x-ray absorptiometry  2) Quadriceps muscle cross-sectional area measured by magnetic resonance imaging  3) Knee extensor muscle strength | 12 weeks | 279 | 15.5 |
| 2 | Painter P (2000) [2] | Physical functioning and health-related quality-of-life changes with exercise training in hemodialysis patients | Quasi-experimental study | 286 (225) | 21.3% | HD | Combined exercise vs. Usual care | 1) Gait speed  2) STS 5  3) 6MWD | 8 weeks | 262 | 10.92 |
| 3 | Kouidi E (1998) [3] | The effects of exercise training on muscle atrophy in haemodialysis patients | Single arm | 7 (7) | 0.0% | HD | Combined exercise | Muscle atrophy | 24 weeks | 238 | 9.15 |
| 4 | Cheema B (2007) [4] | Progressive exercise for anabolism in kidney disease (PEAK): A randomized, controlled trial of resistance training during hemodialysis | RCT | 59 (44) | 10.2% | HD | Resistance training vs. Usual care | Thigh muscle quantity (cross-sectional area [CSA]) and quality (intramuscular lipid content via attenuation) evaluated by computed tomography scan. | 12 weeks | 225 | 13.24 |
| 5 | Manfredini F (2017) [5] | Exercise in Patients on Dialysis: A Multicenter, Randomized Clinical Trial | RCT | 296 (227) | 23.3% | Dialysis | Aerobic exercise (walking exercise) vs. Usual care | 1) 6MWD  2) STS 5 | 24 weeks | 200 | 28.57 |
| 6 | Konstantinidou E (2002) [6] | Exercise training in patients with end-stage renal disease on hemodialysis: Comparison of three rehabilitation programs | Quasi-experimental study | 58 (48) | 17.2% | HD | Supervised outpatient combined exercise vs. Intradialytic combined exercise vs. Unsupervised moderate aerobic exercise vs. Usual care | Cardiorespiratory fitness included VO2peak, heart rate, anaerobic threshold, respiratory exchange ratio. | 24 weeks | 199 | 9.05 |
| 7 | Castaneda C (2001) [7] | Resistance training to counteract the catabolism of a low-protein diet in patients with chronic renal insufficiency - A randomized, controlled trial | RCT | 26 (26) | 0.0% | Pre-dialysis (moderate renal insufficiency) | Low-protein diet plus resistance training vs. Low-protein diet | 1) Total body potassium  2) Mid-thigh muscle area  3) Type I and II muscle-fiber cross-sectional area  4) Protein turnover | 12 weeks | 171 | 7.43 |
| 8 | van Vilsteren MCBA (2005) [8] | The effects of a low-to-moderate intensity pre-conditioning exercise programme linked with exercise counselling for sedentary haemodialysis patients in The Netherlands: results of a randomized clinical trial | RCT | 103 (96) | 6.8% | HD | Combined exercise vs. Usual care | Physical fitness included exercise physical (VO2peak), lower extremity muscle strength (STS 10), manual dexterity and reaction time (Groningen Fitness Test for the Elderly) | 12 weeks | 166 | 8.74 |
| 9 | Castaneda C (2004) [9] | Resistance training to reduce the malnutrition-inflammation complex syndrome of chronic kidney disease | RCT | 26 (26) | 0.0% | Pre-dialysis | Low-protein diet resistance training vs. Low-protein diet | Inflammatory mediators (serum C-reactive protein [CRP] and interleukin-6 [IL-6]) | 12 weeks | 156 | 7.8 |
| 10 | Ouzouni S (2009) [10] | Effects of intradialytic exercise training on health-related quality of life indices in haemodialysis patients | RCT | 35 (33) | 5.7% | HD | Combined exercise vs. Usual care | Cardiorespiratory fitness included VO2peak, heart rate, blood pressure, metabolic equivalents, maximum pulmonary ventilation. | 40 weeks | 152 | 10.13 |
| 11 | Painter P (2002) [11] | Effects of exercise training plus normalization of hematocrit on exercise capacity and health-related quality of life | RCT | 65 (48) | 26.2% | HD | Usual hematocrit (30%–33%) vs. Usual hematocrit (30%–33%)+aerobic exercise vs. Normalized hematocrit (40%–42%) vs. Normalized hematocrit (40%–42%)+aerobic exercise (NHX) | Cardiorespiratory fitness included VO2peak, heart rate, blood pressure, minute ventilation, respiratory exchange ratio. | 20 weeks | 150 | 6.82 |
| 12 | Parsons TL (2006) [12] | Exercise training during hemodialysis improves dialysis efficacy and physical performance | Single arm | 20 (13) | 35.0% | HD | Aerobic exercise | Dialysis efficacy (in single-pool model of urea kinetics [spKt/V]) | 20 weeks | 146 | 8.11 |
| 13 | Painter PL (2002) [13] | A randomized trial of exercise training after renal transplantation | RCT | 167 (97) | 41.9% | KTR | Aerobic exercise vs. Usual care | Cardiorespiratory fitness (VO2peak) | 48 weeks | 144 | 6.55 |
| 14 | Storer TW (2005) [14] | Endurance exercise training during haemodialysis improves strength, power, fatigability and physical performance in maintenance haemodialysis patients | Quasi-experimental study | 12 (12) | 0.0% | HD | Aerobic exercise (stationary leg cycling) vs. Usual care | Cardiorespiratory fitness (VO2peak) | 40 weeks | 139 | 7.32 |
| 15 | Headley S (2002) [15] | Resistance training improves strength and functional measures in patients with end-stage renal disease | Single arm | 10 (10) | 0.0% | HD | Resistance training | 1) Percentage of body fat  2) 6MWD  3) Peak torque of quadriceps muscles of the dominant leg  4) HGS  5) Gait speeds  6) STS 10 | 12 weeks | 138 | 6.27 |
| 16 | Koh KP (2010) [16] | Effect of Intradialytic Versus Home-Based Aerobic Exercise Training on Physical Function and Vascular Parameters in Hemodialysis Patients: A Randomized Pilot Study | RCT | 70 (46) | 34.3% | HD | Home-based aerobic exercise vs. Intradialytic aerobic exercise vs. Usual care | 1) 6MWD  2) Aortic pulse wave velocity | 24 weeks | 134 | 9.57 |
| 17 | Chen JLT (2010) [17] | Effect of intra-dialytic, low-intensity strength training on functional capacity in adult haemodialysis patients: a randomized pilot trial | RCT | 50 (44) | 12.0% | HD | Resistance training vs. Usual care | Physical function (Short Physical Performance Battery) | 24 weeks | 130 | 9.29 |
| 18 | Mustata S (2004) [18] | Impact of an exercise program on arterial stiffness and insulin resistance in hemodialysis patients | Single arm | 16 (11) | 31.3% | HD | Aerobic exercise | 1) Arterial stiffness  2) Insulin resistance | 12 weeks | 123 | 6.15 |
| 19 | Kopple JD (2007) [19] | Exercise in maintenance hemodialysis patients induces transcriptional changes in genes favoring anabolic muscle | Single arm | 80 (51) | 36.3% | HD | Aerobic exercise vs. Resistance training vs. Combined exercise vs. Usual care | mRNA levels of genes in muscle | 21 weeks | 119 | 7 |
| 20 | Yurtkuran M (2007) [20] | A modified yoga-based exercise program in hemodialysis patients: A randomized controlled study | RCT | 40 (37) | 7.5% | HD | Aerobic exercise (yoga) | 1) Pain intensity  2) Fatigue  3) Sleep disturbance  4) HGS | 12 weeks | 116 | 6.82 |
| 21 | Wilund, KR (2010) [21] | Intradialytic exercise training reduces oxidative stress and epicardial fat: a pilot study | RCT | 17 (15) | 11.8% | HD | Aerobic exercise (stationary leg cycling) vs. Usual care | 1) Physical function (Incremental shuttle walk test)  2) Serum parameters related to CVD risk and renal function  3) Variables related to cardiac structure and function | 16 weeks | 113 | 8.07 |
| 22 | Kouidi, E (1997) [22] | Exercise renal rehabilitation program: Psychosocial effects | RCT | 36 (31) | 13.9% | HD | Aerobic exercise (mixed) | 1) Depression  2) Quality of life  3) Personality | 24 weeks | 110 | 4.07 |
| 23 | Koufaki P (2002) [23] | Effects of exercise training on aerobic and functional capacity of end-stage renal disease patients | RCT | 48 (33) | 31.3% | Dialysis | Aerobic exercise (stationary leg cycling) vs. Usual care | Cardiorespiratory fitness (VO2 peak and VO2 at ventilatory threshold) | 12 weeks | 109 | 4.95 |
| 24 | DePaul V (2002) [24] | The effectiveness of aerobic and muscle strength training in patients receiving hemodialysis and EPO: A randomized controlled trial | RCT | 38 (20) | 47.4% | HD | Combined exercise vs. Usual care | Submaximal exercise test | 12 weeks | 108 | 4.91 |
| 25 | Painter PL (1986) [25] | Effects of Exercise Training During Hemodialysis | Quasi-experimental study | 27 (19) | 29.6% | HD | Aerobic exercise (stationary leg cycling) vs. Usual care | Cardiorespiratory fitness (VO2peak) | 24 weeks | 107 | 2.82 |
| 26 | Viana JL (2014) [26] | Evidence for Anti-Inflammatory Effects of Exercise in CKD | Quasi-experimental study | 15 (15) | 0.0% | Pre-dialysis CKD | Aerobic exercise vs. Usual care | 1) Neutrophil degranulatio  2) Activation of T lymphocytes  3) Monocytes  4) Plasma  5) Inflammatory markers | 24 weeks | 106 | 10.6 |
| 27 | Deligiannis A (1999) [27] | Effects of physical training on heart rate variability in patients on hemodialysis | Quasi-experimental study | 90 (90) | 0.0% | HD | Aerobic exercise (mixed) vs. Usual care | Heart rate variability | 24 weeks | 104 | 4.16 |
| 28 | Goldberg AP (1980) [28] | Metabolic Effects of Exercise Training in Hemodialysis-Patients | Single arm | 6 (6) | 0.0% | HD | Aerobic exercise (mixed) | Metabolic index | 24 weeks | 104 | 2.36 |
| 29 | Deligiannis A (1999) [29] | Cardiac effects of exercise rehabilitation in hemodialysis patients | RCT | 53 (53) | 0.0% | HD | Supervised aerobic exercise vs. Home-based aerobic exercise vs. Usual care | Cardiorespiratory fitness included VO2max, heart rate, blood pressure, metabolic equivalents. | 24 weeks | 103 | 4.12 |
| 30 | Greenwood SA (2015) [30] | Effect of Exercise Training on Estimated GFR, Vascular Health, and Cardiorespiratory Fitness in Patients With CKD: A Pilot Randomized Controlled Trial | RCT | 20 (18) | 10.0% | Pre-dialysis CKD 3-4 | Combined exercise vs. Usual care | Kidney function (estimated glomerular filtration rate) | 48 weeks | 102 | 11.33 |


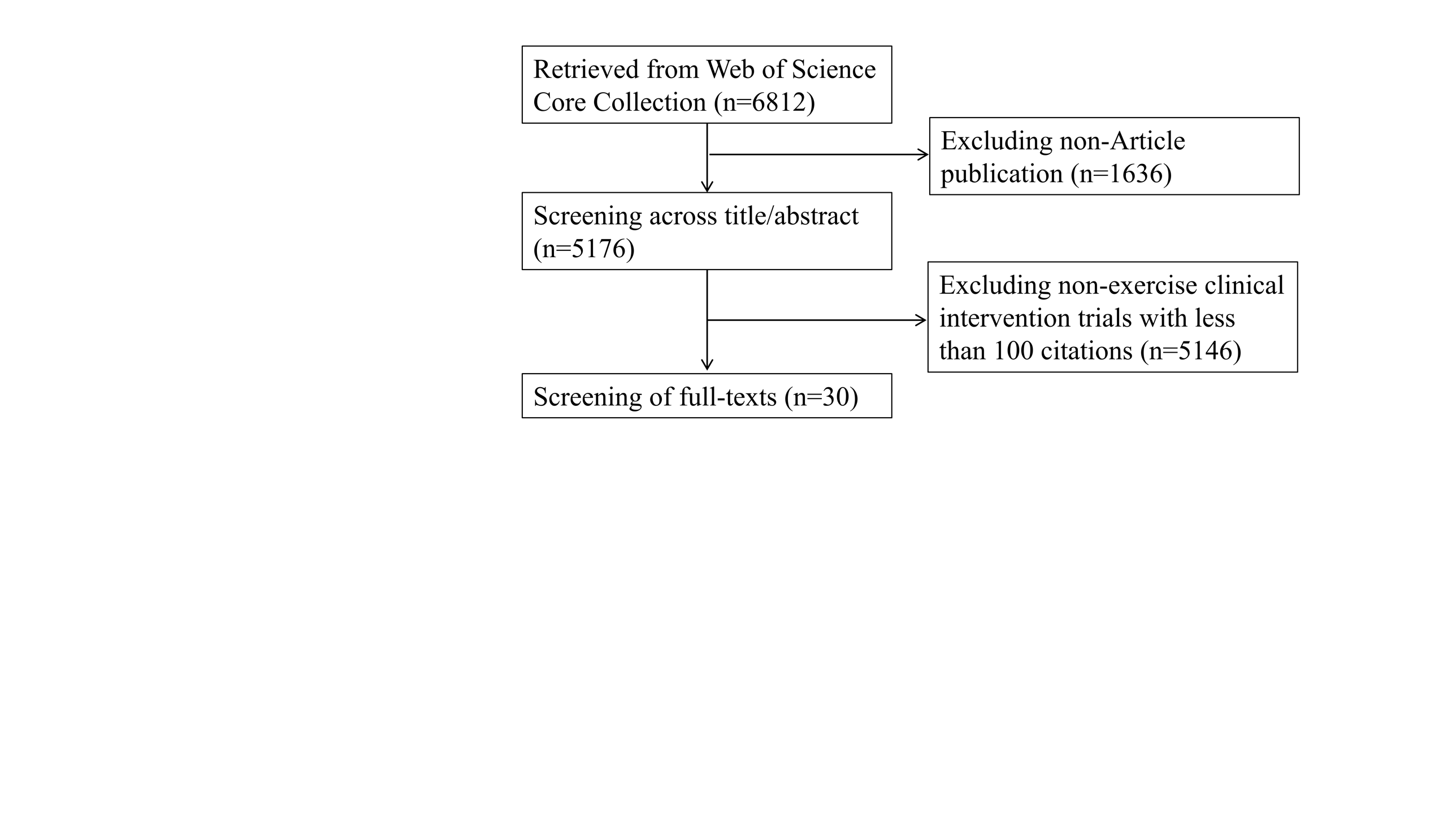


Figure S1 Flow chart of literature screening.


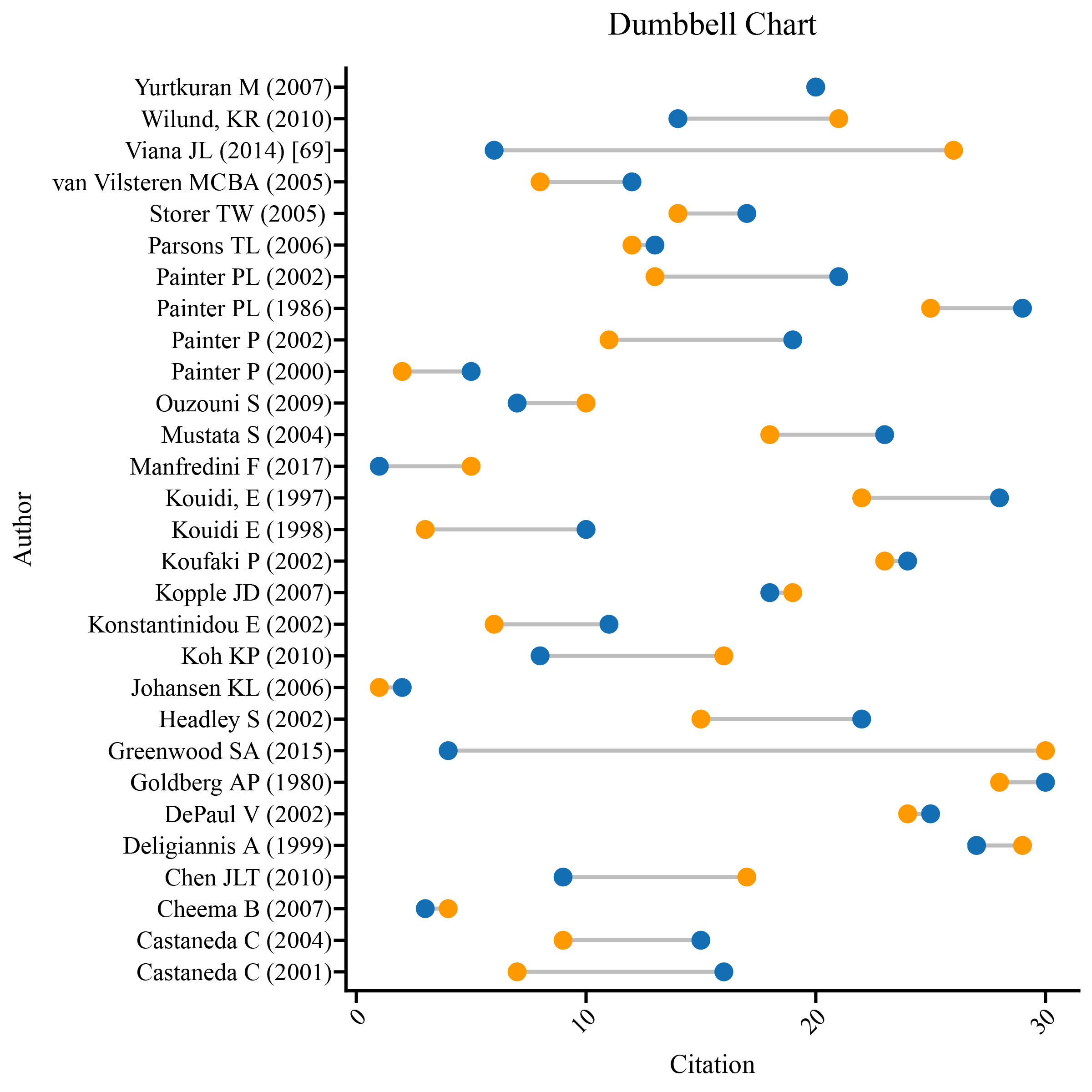


Figure S2 Dumbbell plots of total citations and annual average citations for 30 classic citations

(Note: Yellow circles indicate total citations; blue circles indicate average annual citations)

Reference

1. Johansen KL, Painter PL, Sakkas GK, Gordon P, Doyle J, Shubert T: **Effects of resistance exercise training and nandrolone decanoate on body composition and muscle function among patients who receive hemodialysis: A randomized, controlled trial**. *J Am Soc Nephrol* 2006, **17**(8):2307-2314.

2. Painter P, Carlson L, Carey S, Paul SM, Myll J: **Physical functioning and health-related quality-of-life changes with exercise training in hemodialysis patients**. *AMERICAN JOURNAL OF KIDNEY DISEASES* 2000, **35**(3):482-492.

3. Kouidi E, Albani M, Natsis K, Megalopoulos A, Gigis P, Guiba-Tziampiri O, Tourkantonis A, Deligiannis A: **The effects of exercise training on muscle atrophy in haemodialysis patients**. *NEPHROLOGY DIALYSIS TRANSPLANTATION* 1998, **13**(3):685-699.

4. Cheema B, Abas H, Smith B, O'Sullivan A, Chan M, Patwardhan A, Kelly J, Gillin A, Pang G, Lloyd B *et al*: **Progressive exercise for anabolism in kidney disease (PEAK): A randomized, controlled trial of resistance training during hemodialysis**. *J Am Soc Nephrol* 2007, **18**(5):1594-1601.

5. Manfredini F, Mallamaci F, D'Arrigo G, Baggetta R, Bolignano D, Torino C, Lamberti N, Bertoli S, Ciurlino D, Rocca-Rey L *et al*: **Exercise in Patients on Dialysis: A Multicenter, Randomized Clinical Trial**. *J Am Soc Nephrol* 2017, **28**(4):1259-1268.

6. Konstantinidou E, Koukouvou G, Kouidi E, Deligiannis A, Tourkantonis A: **Exercise training in patients with end-stage renal disease on hemodialysis: Comparison of three rehabilitation programs**. *JOURNAL OF REHABILITATION MEDICINE* 2002, **34**(1):40-45.

7. Castaneda C, Gordon PL, Uhlin KL, Levey AS, Kehayias JJ, Dwyer JT, Fielding RA, Roubenoff R, Singh MF: **Resistance training to counteract the catabolism of a low-protein diet in patients with chronic renal insufficiency - A randomized, controlled trial**. *ANNALS OF INTERNAL MEDICINE* 2001, **135**(11):965-976.

8. van Vilsteren M, de Greef MHG, Huisman RM: **The effects of a low-to-moderate intensity pre-conditioning exercise programme linked with exercise counselling for sedentary haemodialysis patients in The Netherlands: results of a randomized clinical trial**. *NEPHROLOGY DIALYSIS TRANSPLANTATION* 2005, **20**(1):141-146.

9. Castaneda C, Gordon PL, Parker RC, Uhlin KL, Roubenoff R, Levey AS: **Resistance training to reduce the malnutrition-inflammation complex syndrome of chronic kidney disease**. *AMERICAN JOURNAL OF KIDNEY DISEASES* 2004, **43**(4):607-616.

10. Ouzouni S, Kouidi E, Sioulis A, Grekas D, Deligiannis A: **Effects of intradialytic exercise training on health-related quality of life indices in haemodialysis patients**. *CLINICAL REHABILITATION* 2009, **23**(1):53-63.

11. Painter P, Moore G, Carlson L, Paul S, Myll J, Phillips W, Haskell W: **Effects of exercise training plus normalization of hematocrit on exercise capacity and health-related quality of life**. *AMERICAN JOURNAL OF KIDNEY DISEASES* 2002, **39**(2):257-265.

12. Parsons TL, Toffelmire EB, King-Van Vlack CE: **Exercise training during hemodialysis improves dialysis efficacy and physical performance**. *ARCHIVES OF PHYSICAL MEDICINE AND REHABILITATION* 2006, **87**(5):680-687.

13. Painter PL, Hector L, Ray K, Lynes L, Dibble S, Paul SM, Tomlanovich SL, Ascher NL: **A randomized trial of exercise training after renal transplantation**. *TRANSPLANTATION* 2002, **74**(1):42-48.

14. Storer TW, Casaburi R, Sawelson S, Kopple JD: **Endurance exercise training during haemodialysis improves strength, power, fatigability and physical performance in maintenance haemodialysis patients**. *NEPHROLOGY DIALYSIS TRANSPLANTATION* 2005, **20**(7):1429-1437.

15. Headley S, Germain M, Mailloux P, Mulhern J, Ashworth B, Burris J, Brewer B, Nindl BC, Coughlin M, Welles R *et al*: **Resistance training improves strength and functional measures in patients with end-stage renal disease**. *AMERICAN JOURNAL OF KIDNEY DISEASES* 2002, **40**(2):355-364.

16. Koh KP, Fassett RG, Sharman JE, Coombes JS, Williams AD: **Effect of Intradialytic Versus Home-Based Aerobic Exercise Training on Physical Function and Vascular Parameters in Hemodialysis Patients: A Randomized Pilot Study**. *AMERICAN JOURNAL OF KIDNEY DISEASES* 2010, **55**(1):88-99.

17. Chen JLT, Godfrey S, Ng TT, Moorthi R, Liangos O, Ruthazer R, Jaber BL, Levey AS, Castaneda-Sceppa C: **Effect of intra-dialytic, low-intensity strength training on functional capacity in adult haemodialysis patients: a randomized pilot trial**. *NEPHROLOGY DIALYSIS TRANSPLANTATION* 2010, **25**(6):1936-1943.

18. Mustata S, Chan C, Lai V, Miller JA: **Impact of an exercise program on arterial stiffness and insulin resistance in hemodialysis patients**. *J Am Soc Nephrol* 2004, **15**(10):2713-+.

19. Kopple JD, Wang HY, Casaburi R, Fournier M, Lewis MI, Taylor W, Storer TW: **Exercise in maintenance hemodialysis patients induces transcriptional changes in genes favoring anabolic muscle**. *J Am Soc Nephrol* 2007, **18**(11):2975-2986.

20. Yurtkuran M, Alp A, Yurtkuran M, Dilek K: **A modified yoga-based exercise program in hemodialysis patients: A randomized controlled study**. *COMPLEMENTARY THERAPIES IN MEDICINE* 2007, **15**(3):164-171.

21. Wilund KR, Tomayko EJ, Wu PT, Chung HR, Vallurupalli S, Lakshminarayanan B, Fernhall B: **Intradialytic exercise training reduces oxidative stress and epicardial fat: a pilot study**. *NEPHROLOGY DIALYSIS TRANSPLANTATION* 2010, **25**(8):2695-2701.

22. Kouidi E, Iacovides A, Iordanidis P, Vassiliou S, Deligiannis A, Ierodiakonou C, Tourkantonis A: **Exercise renal rehabilitation program: Psychosocial effects**. *NEPHRON* 1997, **77**(2):152-158.

23. Koufaki P, Mercer TH, Naish PF: **Effects of exercise training on aerobic and functional capacity of end-stage renal disease patients**. *Clin Physiol Funct Imaging* 2002, **22**(2):115-124.

24. DePaul V, Moreland J, Eager T, Clase CM: **The effectiveness of aerobic and muscle strength training in patients receiving hemodialysis and EPO: A randomized controlled trial**. *AMERICAN JOURNAL OF KIDNEY DISEASES* 2002, **40**(6):1219-1229.

25. Painter PL, Nelsonworel JN, Hill MM, Thornbery DR, Shelp WR, Harrington AR, Weinstein AB: **Effects of Exercise Training During Hemodialysis**. *NEPHRON* 1986, **43**(2):87-92.

26. Viana JL, Kosmadakis GC, Watson EL, Bevington A, Feehally J, Bishop NC, Smith AC: **Evidence for Anti-Inflammatory Effects of Exercise in CKD**. *J Am Soc Nephrol* 2014, **25**(9):2121-2130.

27. Deligiannis A, Kouidi E, Tourkantonis A: **Effects of physical training on heart rate variability in patients on hemodialysis**. *AMERICAN JOURNAL OF CARDIOLOGY* 1999, **84**(2):197-202.

28. Goldberg AP, Hagberg JM, Delmez JA, Haynes ME, Harter HR: **Metabolic Effects of Exercise Training in Hemodialysis-Patients**. *KIDNEY INTERNATIONAL* 1980, **18**(6):754-761.

29. Deligiannis A, Kouidi E, Tassoulas E, Gigis P, Tourkantonis A, Coats A: **Cardiac effects of exercise rehabilitation in hemodialysis patients**. *INTERNATIONAL JOURNAL OF CARDIOLOGY* 1999, **70**(3):253-266.

30. Greenwood SA, Koufaki P, Mercer TH, MacLaughlin HL, Rush R, Lindup H, O'Connor E, Jones C, Hendry BM, Macdougall IC *et al*: **Effect of Exercise Training on Estimated GFR, Vascular Health, and Cardiorespiratory Fitness in Patients With CKD: A Pilot Randomized Controlled Trial**. *AMERICAN JOURNAL OF KIDNEY DISEASES* 2015, **65**(3):425-434.
